# Experiences of Navigating Recovery After Stroke in Tanzania: A Descriptive Qualitative Study

**DOI:** 10.1155/nrp/1450052

**Published:** 2026-05-16

**Authors:** Evans Luvanda, Rashid Heri, Menti Ndile

**Affiliations:** ^1^ Emergency Medicine Department, Bulongwa Lutheran Hospital, Njombe, Tanzania; ^2^ Department of Nursing Education and Management, Muhimbili University of Health and Allied Sciences, Dar es Salaam, Tanzania, muchs.ac.tz; ^3^ Department of Clinical Nursing, Muhimbili University of Health and Allied Sciences, Dar es Salaam, Tanzania, muchs.ac.tz

**Keywords:** lived experience, qualitative research, rehabilitation, stroke, Tanzania

## Abstract

**Background:**

Stroke is a leading cause of disability globally, with increasing prevalence in sub‐Saharan Africa. Little is known about how Tanzanian survivors navigate recovery within their social and healthcare environments. This study aimed to explore stroke survivors’ lived experiences of barriers to accessing and utilizing poststroke management services at a tertiary hospital in Tanzania.

**Methods:**

A qualitative study using an interpretive descriptive approach was conducted among stroke survivors attending follow‐up care at a tertiary referral hospital in Tanzania. A homogeneous purposive sampling strategy was used to recruit participants who shared key characteristics relevant to the study. The sample comprised 20 stroke survivors, including 8 males and 12 females. In‐depth interviews were conducted and analysed using reflexive thematic analysis to identify patterns of meaning across participants’ experiences.

**Results:**

Four interrelated themes captured survivors’ experiences: (1) living with financial uncertainty, where costs of care and prescribed diets created practical and emotional barriers; (2) making sense of care amid constraints, reflecting adaptive decision‐making and reliance on personal beliefs or home remedies; (3) negotiating healthcare relationships, highlighting family support and the importance of ongoing communication with providers and (4) enduring the healthcare system, where long waiting times, administrative hurdles, frequent provider changes and conditional access disrupted continuity of care.

**Conclusion:**

Stroke recovery in Tanzania extends beyond clinical treatment, which is shaped by intertwined socioeconomic, relational and health system influences. Strengthening discharge planning, enhancing continuity of professional support, integrating family‐centred rehabilitation and leveraging mobile health technologies may improve equitable engagement with stroke care in resource‐constrained settings.

## 1. Introduction

Stroke is a major global public health challenge and remains one of the leading causes of death and long‐term disability worldwide [1]. In 2021, it ranked as the third leading cause of death and disability globally, with an estimated 93.8 million prevalent cases [2]. The burden of stroke is disproportionately concentrated in low‐ and middle‐income countries (LMICs), which account for approximately 87.0% of stroke‐related deaths and 89.0% of disability‐adjusted life years (DALYs) [1]. In Africa, the pooled prevalence of stroke mortality is estimated at 20.3% (95% CI: 17.3–23.2) [3]. In Tanzania, a multicentre study conducted across eight major tertiary hospitals reported a high stroke mortality rate of 31.5% (315 per 1000 patients), with hypertension identified as the leading risk factor [4].

Stroke occurs when the blood supply to the brain is abruptly interrupted, either due to vascular occlusion (ischaemic stroke) or vessel rupture (haemorrhagic stroke), resulting in sudden neurological deficits such as limb weakness, speech impairment, dizziness, visual disturbances, or loss of consciousness [5]. Ischaemic stroke accounts for the majority of incident stroke cases globally, followed by intracerebral haemorrhage [6]. These disruptions in cerebral blood flow lead to neuronal injury and functional impairment [7].

Given its sudden and debilitating nature, effective stroke management requires an integrated continuum of care encompassing rapid emergency recognition and response, timely acute treatment, coordinated inpatient care, structured rehabilitation and long‐term secondary prevention [8]. Evidence from high‐income countries shows that organised stroke systems, including dedicated stroke units and multidisciplinary rehabilitation teams, significantly improve survival, functional recovery and quality of life [9, 10]. However, in many sub‐Saharan African settings, the delivery of comprehensive stroke care remains constrained by limited infrastructure, shortages of trained specialists, fragmented rehabilitation services and financial barriers. These systemic challenges hinder continuity of care and contribute to poorer outcomes, increased disability and substantial psychosocial and economic burdens for survivors and their caregivers [11, 12].

A recent qualitative study conducted in Tanzania identified multiple systemic challenges across the stroke care continuum, including limited health literacy among survivors, the significant financial burden associated with stroke services and shortages of essential resources at lower‐level health facilities [13]. Although the study’s multistakeholder design generated important system‐level insights, it may not have sufficiently captured the depth and complexity of stroke survivors’ lived experiences. Focusing exclusively on survivors’ lived experiences allows for a deeper exploration of how barriers are personally encountered, interpreted and managed daily, a crucial perspective for designing patient‐centred interventions that address not only clinical needs but also the psychosocial and economic realities of recovery. Therefore, this study explored stroke survivors’ lived experiences of barriers to access and utilise poststroke management services at a tertiary hospital in Tanzania, capturing both individual and systemic factors and how survivors interpret and navigate these challenges in their daily recovery.

## 2. Materials and Methods

### 2.1. Study Design

This study employed an interpretive descriptive qualitative design, guided by an interpretivist paradigm, to explore stroke survivors’ lived experiences of barriers to access and utilise poststroke management services at a tertiary hospital in Tanzania [14]. The interpretivist paradigm assumes that experiences are socially and contextually constructed, and that knowledge is cocreated through interactions between the researcher and participants [15]. This approach enabled stroke survivors to express, in their own words, how they encountered, interpreted and managed barriers when accessing and utilising poststroke management services. It also supported an in‐depth interpretive analysis, allowing the research team to generate rich, nuanced insights into the factors influencing access to care. The study adheres to the Standards for Reporting Qualitative Research (SRQR) [16] to ensure comprehensive and transparent reporting (Supporting File 1).

### 2.2. Study Setting

This study was conducted at Muhimbili National Hospital (MNH)‐Mloganzila, a leading tertiary referral hospital in Tanzania. As a major public facility, it offers specialized stroke services, including diagnostic imaging, intensive care, neurology and rehabilitation. Given its role in serving a diverse patient population from across the country, MNH‐Mloganzila provides an ideal setting for exploring the experiences of stroke survivors in accessing and utilising stroke management services.

### 2.3. Study Population and Sampling

Purposive sampling was used to recruit stroke survivors who had received poststroke care at MNH‐Mloganzila [17]. To ensure a focus on the shared essence of the lived experience, a homogeneous sampling strategy was employed. Participants were selected based on common criteria: All were first‐time stroke survivors, aged 18 years or older, between three and 12 months poststroke and capable of articulating their experiences. This ensured that variations in the phenomenon could be attributed to the experience of barriers itself, rather than to differences in clinical or demographic characteristics. A total of 20 participants were enrolled; data saturation was reached by the 18th interview, with two additional interviews conducted to confirm this.

### 2.4. Data Collection Tool and Procedure

A semistructured interview guide was developed based on a review of the relevant literature to explore stroke survivors’ lived experiences of barriers to accessing and utilising poststroke management services. To avoid constraining participants with prior assumptions, questions were framed openly (e.g., “Can you describe your experiences accessing and using stroke care services after your stroke?”), followed by probing questions, allowing participants to express their perspectives in their own terms. The guide was pilot‐tested with three participants, and minor refinements to question wording and sequencing were made.

Potential participants were approached during routine hospital visits. The study purpose and procedures were explained, and preliminary consent was sought. Interviews were scheduled at the participants’ convenience to minimize disruption.

Data collection occurred between 7th and 30th May 2025 in a private hospital room to ensure confidentiality. Written informed consent was obtained before each interview. Interviews were conducted in Swahili, lasting 60–90 min. Insights from early interviews informed iterative adjustments to the guide, ensuring continued relevance and responsiveness to participants’ narratives. With consent, interview sessions were audio‐recorded and supplemented with field notes capturing nonverbal cues, context and researcher reflections. All data were anonymised and securely stored to maintain confidentiality.

### 2.5. Data Analysis

The analysis followed the six‐phase process of reflexive thematic analysis, as described by Braun and Clarke [18]. To ensure authenticity to participants’ voices, EL first transcribed all Swahili recordings verbatim. Working in the original language, EL performed detailed line‐by‐line coding to capture initial concepts without the filter of translation. These codes were then discussed collectively with the supervisory team (MN and RH), and collaborative translation into English was undertaken. This process emphasised conceptual accuracy, ensuring that the intended meanings were maintained throughout.

The team engaged in regular, iterative meetings to review emerging patterns. During these sessions, codes were clustered and themes were proposed and challenged. To strengthen analytical rigour, ongoing team discussions with active supervisory input from MN and RH established the coherence and credibility of the thematic structure. An external qualitative research expert was also invited to review the analysis, offering an independent interpretive insight that further enhanced the dependability of the findings. Direct quotations from participants were purposefully selected to vividly illustrate each theme in the final report.

### 2.6. Reflexivity

Throughout the research process, the team engaged in ongoing critical reflection on how their professional backgrounds might shape interactions with participants and the interpretation of the data. EL, a postgraduate cardiovascular nursing student, conducted the interviews and was primarily responsible for the analysis. Supervisory support was provided by MN, whose experience spans critical care nursing and education, and RH, also a nurse educator. The team’s collective clinical grounding in cardiovascular and critical care contexts proved valuable in establishing rapport with participants and understanding the clinical context of their narratives.

Participants were recruited from MNH‐Mloganzila, where EL had prior institutional familiarity but no direct clinical or personal relationship with the participants. This positioning facilitated access to the study setting while helping maintain a degree of independence during data collection. At the same time, the shared healthcare environment may have influenced participants’ perceptions of the researcher, potentially shaping their responses.

To mitigate this, EL emphasized his role as a researcher rather than a care provider and clarified that participation would not affect the care participants received. A deliberate approach of bracketing was adopted during interviews, with EL consciously setting aside prior clinical knowledge and assumptions to remain attentive to participants’ subjective experiences.

Analytical meetings involving all three team members provided a further layer of critical scrutiny. During these sessions, interpretations were routinely examined, questioned and compared against the data to ensure that emerging themes reflected participants’ meanings rather than professional presuppositions.

Attention to ethical complexities and power relations remained central throughout. The team prioritized participants’ autonomy by reinforcing the voluntary nature of participation and ensuring that consent was treated as an ongoing process. Confidentiality was strictly maintained, and all interactions were conducted with sensitivity and respect. These reflexive practices supported the trustworthiness of the study and ensured that the findings authentically represented stroke survivors’ lived experiences of barriers to care.

### 2.7. Trustworthiness

Trustworthiness was ensured in line with interpretive phenomenological rigor. Credibility was supported through in‐depth, semistructured interviews that enabled participants to articulate their lived experiences, alongside iterative probing and ongoing interpretive engagement with the data. Initial findings were shared with a subset of participants to validate interpretations and ensure that the emerging meanings resonated with their experiences. Regular analytic discussions among the research team facilitated the coconstruction and critical examination of meanings. Dependability was enhanced by maintaining a transparent audit trail of methodological and analytical decisions. Confirmability was addressed through reflexive practices, including critical self‐reflection and team‐based analysis, acknowledging the researchers’ interpretive role while grounding the findings in participants’ accounts. Transferability was facilitated through rich, contextual descriptions of the study setting, participants and findings, allowing readers to assess applicability to similar contexts.

### 2.8. Ethical Considerations

Ethical clearance was obtained from the Research and Ethics Committee of the Muhimbili University of Health and Allied Sciences (Ref. No. DA.282/298/D1.C/2824). Following this, formal permission to conduct the study at the hospital was granted by the hospital management. All participants provided written informed consent before taking part in the study. The consent form clearly outlined the voluntary nature of participation, the right to withdraw at any time without consequence, potential risks and benefits and the procedures for audio recording interviews. To safeguard participant identity, all data were anonymised, and strict confidentiality was maintained throughout the research process.

## 3. Results

### 3.1. Sociodemographic Characteristics

A total of 20 stroke survivors participated in the study. As presented in Table 1, the majority of participants were middle‐aged adults aged 36–55 years (*n* = 13, 65%), and more than half were female (*n* = 12, 60%). Regarding educational attainment, most participants (*n* = 14, 70%) had a college‐level qualification (certificate or diploma), while a smaller proportion held university degrees (*n* = 2, 10%).

**TABLE 1:** Sociodemographic characteristics of stroke survivors (*N* = 20).

Characteristic	Frequency (*n*)	Percentage (%)
Age (Years)		
Young adults (18–35)	2	10
Middle‐aged (36–55)	13	65
Older adults (56+)	4	25
Sex		
Male	8	40
Female	12	60
Level of education		
Primary and secondary level	4	20
College‐level qualification	14	70
University degree	2	10
Marital status		
Single	5	25
Married	10	50
Widow/widower	3	15
Divorced	2	10
Stroke survival duration		
3–6 months	9	45
7–12 months	11	55

Half of the participants were married (*n* = 10, 50%), whereas the remaining participants were single, widowed or divorced. In terms of stroke survival duration, most survivors had lived with stroke for between 7 and 12 months (*n* = 11, 55%).

The analysis revealed that stroke recovery is a complex lived process that extends beyond medical treatment into everyday challenges involving financial survival, emotional endurance, relational negotiation and navigation of healthcare systems. Four interrelated themes captured how survivors experienced and interpreted their engagement with stroke management services (Table 2).

**TABLE 2:** Themes, subthemes and interpretive codes.

Theme	Subtheme	Interpretive codes
1. Living with financial uncertainty	Financial strain and feelings of uncertainty	• Hopelessness due to inability to afford the recommended diet • Sorrow over life without financial protection • Out‐of‐pocket payment transforming care into a survival negotiation
	Emotional weight of stroke care costs	• Anxiety, helplessness, and sadness linked to financial strain • Fear of inability to sustain treatment discourages care‐seeking • Emotional burden of calculating survival costs for medication, therapy and transport

2. Making sense of care amid constraints	Beliefs and knowledge gaps	• Negative attitudes towards rehabilitation shaped by perceived impracticality • Misunderstanding of stroke recovery processes contributing to hesitation • Preference for traditional or home‐based remedies as rational, culturally familiar options
	Practical decision‐making	• Choosing affordable, accessible options over recommended hospital care • Balancing cultural familiarity with economic constraints • Adapting care routines to fit personal, family and financial realities

3. Negotiating healthcare relationships	Communication and access to providers	• Desire for contact with healthcare providers when symptoms arise • Frustration and insecurity when doctors refuse phone access • Requests for institutional solutions: on‐call doctors or facility hotlines
	Family and social support dynamics	• Dependence on family constrained by household financial limits • Lack of peer or family encouragement undermining rehabilitation adherence • Family conflict over treatment choices (traditional vs. hospital care) • Emotional tension between gratitude for support and fear of burdening others

4. Enduring the healthcare system	Challenges with timing and processes	• Long waiting times, overcrowded clinics and multistep registration processes • Exhaustion, discouragement and missed care due to procedural delays • Waiting experienced as temporal violence against weakened bodies
	Continuity and relational disruption	• Frequent doctor changes disrupting trust and therapeutic momentum • Need to repeatedly recount medical history • Fragmentation of care experienced as abandonment or neglect
	Conditional access and institutional limitations	• Treatment contingent on financial capacity rather than medical need • Insurance gaps, uncovered services and prior hospital debts limiting care • Organisational structures amplifying survivors’ sense of vulnerability and dependence

## 4. Living With Financial Uncertainty

### 4.1. Financial Strain and Feelings of Uncertainty

Financial hardship emerged as a significant and underlying influence shaping survivors’ recovery experiences. Participants described stroke not only as a health condition but as an economic crisis that disrupted stability, independence and future security. Treatment costs extended beyond consultation fees to include medications, diagnostics, transport, physiotherapy and prescribed dietary changes. Even insured participants experienced gaps in coverage requiring repeated out‐of‐pocket payments.

One participant explained:

*“Life is difficult for me; I don’t have insurance; I pay for everything out of pocket.”* (P9, Age 46)

Another shared the emotional shock following the loss of insurance support:

*“After my insurance expired, I started to worry all the time… it was painful to think that I might not get better because of money.”* (P16, Age 52)

Participants often described financial vulnerability as a sense of abandonment or loss of protection:

*“Living without insurance feels like being left alone… now I can only wait and hope.”* (P12, Age 34)

Recommended dietary changes created additional financial strain. One participant noted:

*“You’re told to use certain foods… but some of those things are expensive.”* (P15, Age 42)

Stroke care, therefore, became a continuous negotiation between health needs and financial capacity, transforming treatment decisions into survival decisions.

### 4.2. Emotional Weight of Stroke Care Costs

Financial difficulties were closely associated with emotional challenges, as participants described feelings of anxiety, sadness and helplessness arising from uncertainty about continuing treatment. Fear of unaffordable care discouraged hospital attendance. One participant shared:

*“I stayed home because I could not afford the cost of going to the hospital. It made me feel helpless.”* (P10, Age 55)

Participants explained experiencing emotional exhaustion from constantly calculating whether they could afford medication refills, transport or therapy sessions. Financial distress diminished motivation for recovery and fostered feelings of hopelessness about long‐term improvement.

These narratives demonstrate that financial hardship functioned not only as a material but also as a psychological barrier, shaping care‐seeking behaviour.

## 5. Making Sense of Care Amid Constraints

### 5.1. Beliefs and Knowledge Gaps

Participants interpreted their stroke experiences through personal beliefs, past experiences and varying levels of understanding about recovery. For some, this included underestimating stroke severity or holding misconceptions about rehabilitation, which influenced when and how they engaged with available services.

Financial pressures and uncertainty often shaped decisions about rehabilitation. One participant explained:

*“They told me to go for physiotherapy every week, but where will I get that money? I started thinking it doesn’t help anyway.”* (P7, Age 45)

In many cases, traditional or home‐based remedies were chosen as culturally familiar and more affordable alternatives. Another participant narrated:

*“I decided to use herbs from a local healer. At least it doesn’t require money like the hospital.”* (P3, Age 59)

These accounts illustrate that participants’ choices were not simply a matter of ignorance. Instead, they reflect meaningful attempts to interpret their condition and regain a sense of control within the constraints of their knowledge, culture and resources.

### 5.2. Practical Decision‐Making

Survivors also demonstrated practical reasoning in navigating care, balancing clinical advice with the realities of daily life. Treatment decisions were influenced by affordability, family responsibilities, travel distance and emotional readiness.

Participants often modified care routines, delayed hospital visits or adopted home‐based exercises as adaptive strategies. One participant shared:

*“I try to do some of the exercises at home because going to the clinic every week is too expensive.”* (P8, Age 50)

These practices show that care engagement reflected pragmatic adaptation rather than passive noncompliance, highlighting survivors’ agency in managing recovery within real‐world constraints.

## 6. Negotiating Healthcare Relationships

### 6.1. Communication and Access to Providers

Participants highlighted the importance of ongoing communication with healthcare providers after discharge. Many expressed fear and uncertainty when symptoms changed at home without professional guidance. One participant suggested:

*“They should give phone numbers to patients so that we can get help while at home.”* (P14, Age 48)

Another participant described the anxiety experienced when symptoms worsened:

*“Sometimes my condition worsens… I experience new symptoms… without the doctor’s contact, I don’t know how to manage.”* (P11, Age 42)

Participants also proposed institutional solutions, such as on‐call doctors or accessible consultation lines, reflecting a strong desire for continuity of care beyond hospital encounters.

Despite these communication challenges, many survivors shared appreciation for clinicians’ compassion and support, demonstrating ongoing efforts to maintain trust and collaborative relationships even within system constraints.

### 6.2. Family and Social Support Dynamics

Family members played a central role in stroke recovery, providing transport, financial assistance and daily care. Many survivors relied entirely on relatives to access hospital services.

However, financial strain within households sometimes limited sustained support. One participant explained:

*“My husband was already in debt from my hospital stay… so I just stayed at home.”* (P13, Age 67)

A lack of encouragement also affected treatment adherence. Another participant shared:

*“No one reminded me or asked how I was doing… I felt alone, so I stopped going for therapy.”* (P18, Age 52)

Family disagreements occasionally disrupted care continuity. One participant described:

*“My children wanted me to use local herbs, but the doctor said no. We argued, and I stopped treatment for weeks.”* (P5, Age 46)

Participants’ narratives reflected a tension between gratitude for support and concern about becoming a burden, highlighting the emotional complexity of dependence during recovery.

## 7. Enduring the Healthcare System

### 7.1. Challenges With Timing and Processes

Long waiting times were commonly reported by participants. Many explained that even when they arrived early, services were often delayed due to overcrowding and staff shortages. One participant shared:

*“Sometimes I spend almost the whole day just waiting.”* (P8, Age 44)

Administrative procedures and technological challenges added further strain. Another participant described:

*“The computer system doesn’t work well… it makes you wait longer and leaves you exhausted.”* (P17, Age 50)

For survivors who were physically weakened, waiting itself became an exhausting and discouraging experience, affecting both motivation and capacity to engage consistently with care.

### 7.2. Continuity and Relational Disruption

Frequent changes of healthcare providers often disrupted therapeutic relationships and rehabilitation progress. One participant explained:

*“When I’m assigned a different doctor, it feels like I have to start explaining myself all over again.”* (P6, Age 35)

Participants highlighted the value of continuity, noting that familiar clinicians were better able to understand their recovery journey. Disruptions in care often generated feelings of insecurity and slowed the momentum of rehabilitation, affecting both confidence and engagement in the recovery process.

### 7.3. Conditional Access and Institutional Limitations

Access to stroke management services was frequently shaped by financial requirements rather than medical urgency. One participant explained:

*“I have to pay part of the debt first before I can receive any care.”* (P1, Age 47)

Limitations of insurance coverage, uncovered services and high medication costs further constrained participants’ ability to engage consistently with treatment. Another participant noted:

*“If I had insurance, I would do physiotherapy regularly.”* (P2, Age 75)

Participants’ economic instability, including unemployment and reliance on small businesses or family support, intensified their vulnerability within the healthcare system, making care access a continuous negotiation between need and means.

## 8. Discussion

This study explored the lived experiences of stroke survivors in Tanzania, revealing how financial, cognitive, relational and systemic factors collectively influence recovery pathways. The findings indicate that survivors’ engagement with care is shaped not only by clinical needs but also by the interaction of economic challenges, limited knowledge, social relationships and constraints within the healthcare system.

Financial hardship emerged as a central influence affecting both practical recovery processes and emotional wellbeing. Participants described stroke not only as a health event but also as an economic disruption requiring continuous expenditure on medications, diagnostics, transport, physiotherapy and dietary modifications. Even among insured individuals, incomplete coverage resulted in repeated out‐of‐pocket payments, reflecting persistent financing gaps reported across African rehabilitation systems [11, 19]. These economic demands limited consistent service utilisation and reinforced inequalities in access to long‐term rehabilitation, a challenge widely documented in low‐resource settings where stroke care remains fragmented and rehabilitation services are scarce [12, 20].

Beyond material constraints, financial insecurity carried substantial psychological consequences. Survivors expressed anxiety, sadness and uncertainty related to sustaining treatment, and fear of unaffordable care sometimes discouraged hospital attendance. Similar associations between economic strain, depressive symptoms and reduced adherence to rehabilitation have been reported across sub‐Saharan Africa [11]. The findings therefore suggest that financial hardship functions simultaneously as a structural and emotional barrier, shaping motivation, hope and perceived recovery potential.

Survivors’ understanding of stroke and recovery further shaped their engagement with care. Participants interpreted their experiences through personal beliefs, previous health encounters and differing levels of knowledge about rehabilitation processes. Limited awareness of stroke severity and realistic recovery timelines occasionally contributed to delayed utilisation of formal healthcare services, a finding consistent with studies conducted in Tanzania and Uganda [13, 21]. Financial pressures often intersect with these knowledge gaps, encouraging reliance on culturally familiar or more affordable home‐based remedies. Rather than indicating disengagement, these responses reflect adaptive efforts by survivors to manage illness in ways that were meaningful and feasible within their cultural context and available resources.

Professional guidance emerged as a central factor influencing recovery experiences. Participants emphasised the importance of clear, consistent communication with healthcare providers, particularly after hospital discharge, when they assumed greater responsibility for self‐management. Limited access to professional support during periods of symptom change often generated uncertainty, anxiety and emotional distress, highlighting the need for structured follow‐up mechanisms such as telephone consultations or community‐based support services. Similar gaps in postdischarge continuity have been reported within Tanzanian stroke care pathways, where survivors and caregivers frequently navigate recovery with minimal ongoing professional guidance. In response to these challenges, recent evidence indicates a growing adoption of mobile health (mHealth) interventions to support stroke survivors following discharge. These digital approaches can be tailored to individual patient needs and have demonstrated potential to strengthen continuity of care through structured telephone communication and remote follow‐up with healthcare providers, thereby improving recovery outcomes and access to rehabilitation services [22, 23]. Importantly, Tanzania is well‐positioned to benefit from such approaches, given the widespread availability of mobile network operators and the high level of mobile phone ownership among adults, which provides a practical foundation for scalable, cost‐effective mHealth‐supported stroke follow‐up programs.

Family members constituted the primary source of practical and emotional assistance, providing transportation, financial resources and daily caregiving. However, financial strain within households and occasional interpersonal tensions sometimes complicated treatment adherence. Survivors described balancing gratitude for support with concerns about dependency and burden, reflecting the emotional complexity of poststroke adjustment. Evidence indicates that limited social support is a significant predictor of poststroke depression [24], while stronger social networks are associated with fewer depressive symptoms, lower disability levels and improved rehabilitation motivation among survivors in Tanzania and other settings [25, 26]. These findings underscore the dual role of families as both facilitators of recovery and participants in shared vulnerability.

System‐level challenges further influenced survivors’ engagement with care. Long waiting times, administrative procedures, technological barriers and frequent changes in healthcare providers created fatigue and diminished confidence in services. Access to treatment was often experienced as contingent upon financial clearance rather than medical urgency, reinforcing vulnerability among economically disadvantaged patients. Comparable systemic constraints—including workforce shortages, infrastructural limitations and fragmented rehabilitation pathways have been widely reported across African health systems [11, 20]. Importantly, the study demonstrates that variations in care engagement cannot be interpreted solely as individual motivation but must be understood as outcomes of ongoing negotiation within structural, economic and institutional constraints.

Taken together, this study extends the current understanding of stroke recovery in sub‐Saharan Africa by demonstrating that survivors’ engagement with rehabilitation is not determined solely by clinical severity or individual motivation but emerges through continuous negotiation within economic, relational and health system environments. While previous research has identified financial barriers, limited knowledge and service fragmentation independently, the present findings highlight how these factors interact across the recovery trajectory, particularly during the postdischarge period when professional support diminishes, and responsibility shifts to survivors and families. The study, therefore, reframes recovery as a socially embedded and context‐dependent process shaped by structural vulnerability as much as by personal resilience. Importantly, Tanzania’s expanding mobile communication infrastructure presents a realistic opportunity to bridge continuity‐of‐care gaps through low‐cost mHealth‐supported follow‐up, suggesting a feasible pathway for strengthening rehabilitation systems without requiring extensive new infrastructure. These findings support the need for integrated stroke care models that combine financial protection, structured discharge planning, caregiver‐inclusive education and technology‐enabled professional follow‐up.

### 8.1. Implications for Practice and Policy

The findings highlight several practical opportunities to strengthen stroke care services within Tanzania and similar low‐resource settings. First, structured discharge planning should be prioritized to ensure survivors receive clear guidance on medication use, rehabilitation expectations, warning signs and available support services before leaving the hospital. Second, continuity of professional support could be improved through nurse‐led or multidisciplinary follow‐up models, including scheduled telephone consultations or mHealth communication, which are feasible given the high level of mobile phone ownership and expanding telecommunications coverage in Tanzania. Third, integrating financial protection mechanisms into chronic stroke care, such as expanded insurance coverage for rehabilitation, may reduce treatment interruption caused by economic hardship. Finally, family‐centred rehabilitation approaches that include caregiver education and psychosocial support are essential, recognizing that families are central partners in recovery rather than informal substitutes for formal care. Collectively, these strategies may promote more equitable access to rehabilitation, enhance patient engagement and improve long‐term functional outcomes following stroke.

### 8.2. Strength and Limitations

This study provides an in‐depth exploration of stroke survivors’ lived experiences within the Tanzanian healthcare context, highlighting how financial, cognitive, relational and systemic factors collectively shape recovery. A major strength lies in the interpretive qualitative approach, which enabled exploration beyond observable barriers to capture survivors’ meanings of recovery and care engagement. The inclusion of participants with diverse sociodemographic backgrounds enhanced the breadth of perspectives, while grounding findings in real‐world clinical and social contexts strengthens their relevance for patient‐centred rehabilitation in low‐resource settings.

However, several limitations should be acknowledged. The study was conducted at a single tertiary referral hospital, which may limit transferability to rural or lower‐level healthcare settings. Participants were those able to engage in interviews, potentially underrepresenting individuals with severe disabilities or those disengaged from care. Although qualitative findings are not intended for statistical generalization, detailed contextual descriptions support applicability to similar contexts. Additionally, reliance on retrospective accounts may introduce recall bias or evolving interpretations of recovery experiences over time.

Furthermore, the researchers’ clinical backgrounds and prior familiarity with the study setting may have influenced both data collection and interpretation. While efforts such as bracketing, reflexive discussions and team‐based analysis were employed to minimize bias, it is possible that participants’ responses and the framing of findings were shaped, to some extent, by shared professional and institutional contexts.

## 9. Conclusion

Stroke recovery in Tanzania extends far beyond physical rehabilitation, unfolding within a complex interplay of financial vulnerability, evolving knowledge, family dynamics and health system structures. Survivors actively navigate recovery by adapting to constrained resources while striving to maintain independence and hope. The findings highlight that meaningful engagement with stroke care depends not only on medical treatment but also on continuity of professional guidance, economic protection and supportive social environments. Strengthening discharge transitions, integrating family‐centred rehabilitation and leveraging accessible mobile health technologies offer practical pathways towards more equitable and sustainable stroke recovery in resource‐constrained settings. Recognizing recovery as a socially embedded process is essential for designing patient‐centred stroke services that align with the lived realities of survivors.

## Author Contributions

Evans Luvanda was involved in the study conception, design, data collection, data analysis and manuscript writing.

Rashid Heri was involved in the study design, data analysis, manuscript writing and revision.

Menti Ndile was involved in the study design, data analysis, manuscript writing and revision.

## Funding

No funding was received for this manuscript.

## Disclosure

All authors read and approved the final manuscript.

## Conflicts of Interest

The authors declare no conflicts of interest.

## Supporting Information

Additional supporting information can be found online in the Supporting Information section.

## Supporting information


**Supporting Information 1** SI Text: Standards for Reporting Qualitative Research (SRQR) checklist.

**Supporting Information 2** S2 Text: Interview guide.

## Data Availability

The data that support the findings of this study are available from the corresponding author upon reasonable request.
