# Supplementary material for: Experiences of Navigating Recovery After Stroke in Tanzania: A Descriptive Qualitative Study
Source: Nurs Res Pract. 2026 May 16;2026:1450052. doi: 10.1155/nrp/1450052 (PMC13179747; doi:10.1155/nrp/1450052)
Supplement: Supplementary file 2 — Supporting Information 2 S2 Text: Interview guide. [file NRP-2026-1450052-s001.docx]

**Interview guide on experiences of navigating recovery after stroke in Tanzania**

**PART A: Background Information**

1. Age
2. Sex
3. Educational background
4. Marital status
5. Time since stroke diagnosis

**PART B: Interview questions**

1. Can you tell me about your experience when you first had a stroke?
2. How did the stroke affect your social and economic situation?
3. How do you decide what helps you recover or stay healthy after a stroke?
4. Can you describe your relationship with healthcare providers since your stroke?
5. What has your experience been like when seeking care after discharge?

**END**
